# Electrochemical Synthesis of Superhydrophobic Polyaniline/Silane Coating Towards Corrosion Protection of Mild Steel

**DOI:** 10.3390/polym18161962

**Published:** 2026-08-11

**Authors:** Yu Chen, Haoyao Zhou, Niteng Fang

**Affiliations:** 1 School of Intelligent Manufacturing, Zhejiang Guangsha Vocational and Technical University of Construction, Jinhua 322100, China; 2 School of Materials and Energy, Foshan University, Foshan 528000, China

**Keywords:** mild steel, corrosion protection, superhydrophobic, polyaniline, silane

## Abstract

In this study, a two-step electrodeposition strategy was proposed to fabricate an eco-friendly superhydrophobic polyaniline (PANI)/silane composite coating on mild steel for corrosion protection. The first step consists of creating a pure PANI adherent coating using a cyclic voltammetry technique and the second step consists of realizing superhydrophobic features using electrodeposition in mixed silane monomers at constant potential. The structure and composition characterization results revealed successful modification of the underlying pure PANI layer with superhydrophobic surface silane film. Wettability tests indicated a high contact angle of 155° and a low sliding angle of 4.2°. Electrochemical measurements revealed that the corrosion current density of PANI/silane-coated mild steel decreased by approximately three orders of magnitude compared to the uncoated sample and the corrosion protection efficiency was as high as 99.6%. Moreover, the prepared PANI/silane hybrid coating exhibited good chemical stability and strong adhesion after 10 days of corrosion in 3.5 wt.% NaCl solution.

## 1. Introduction

Conductive polymers have been widely used to protect metal materials against corrosion in harsh environments due to their exceptional corrosion resistance [1,2,3,4]. The excellent anti-corrosion ability of conductive polymers is due to many factors, such as surface passivation, physical shielding and inhibiting electron flow [5]. Polyaniline (PANI), as a classic conductive polymer, has attracted widespread attention in the corrosion protection field due to its facile and economical fabrication and environmental friendliness [6,7,8]. PANI coatings can be fabricated using various strategies, such as electropolymerization [9,10], chemical polymerization [11,12], photochemical polymerization [13], and so on. Among these methods, electrochemical polymerization is the most popular because of its simplicity and the fact that it allows for easy control of coating thickness and composition [14]. Amegroud [15] prepared a protective PANI coating on a nickel–aluminum bronze alloy substrate using a galvanostatic electropolymerization method. The deposited PANI coating operated as a mixed-type inhibitor and the inhibition efficiency was about 93%. ${PO}_{4}^{-}$- and ${NO}_{3}^{-}$-doped PANI coatings were electrochemically deposited onto 904L stainless steel to inhibit localized corrosion behavior [16]. ${PO}_{4}^{-}$-doped PANI exhibited slightly higher protection efficiency than the ${NO}_{3}^{-}$-doped PANI coating, which may be ascribed to an enhanced passivation effect of the underlying 904L stainless steel by PANI-PO_4_. The good anti-corrosion abilities of these two coatings originated from the strengthened passive layer at the stainless-steel substrate–solution interface along with their physical barrier effect. Nevertheless, their relatively weak adhesion and insufficient long-term corrosion protection properties significantly limit the broad application of pure PANI coatings in practical environments.

To enhance the lifetime of pure PANI layers, a multi-layer coating architecture composed of an underlying PANI layer and a suitable surface film is a possible strategy. It is known that superhydrophobic silanization treatment is a critical strategy to restrain corrosion degradation through reducing the interaction between aggressive electrolytes and metallic surfaces [17,18,19,20]. Ju [21] fabricated an eco-friendly superhydrophobic silane film on a NdFeB substrate through electrochemically assisted deposition. The corrosion current density was reduced by 71.5%, whereas the magnetic characteristics of the sintered NdFeB substrate were almost unchanged. Shen [22] designed a superhydrophobic F-SiO_2_@PDMS composite coating via a two-step spraying method with a high contact angle of 153.2°. The EIS results suggested that the impedance modulus increased by approximately three orders of magnitude compared to the blank sample. Fan [23] prepared a superhydrophobic polysiloxane coating on a carbon steel substrate by a hydrothermal method and subsequent self-assembly. The obtained superhydrophobic coating increased the noise resistance by five times and decreased the standard deviation of the current noise by 40%, exhibiting a good anti-corrosion ability. Han [24] constructed a stable superhydrophobic coating on an Al alloy surface by implanting TiO_2_/SiO_2_ into a silane composite film, which showed high adhesion strength, strong stability and good corrosion protection properties. However, the proposed methods usually require expensive equipment or involve complex production procedures. Moreover, some approaches use fluorinated chemicals, which are harmful to the environment.

Some researchers have reported the combination of a PANI layer and silane film to prevent metal materials from corrosion in different environments [25,26]. For example, ref. [25] prepared fluorinated PANI/GPTMS hybrid films via a sol–gel method. The obtained composite films showed a good corrosion protective ability. Ali [27] reported a novel tri-layer composed of Ni, PANI and a silane based on a sol–gel layer to improve the corrosion protection performance of AZ91 magnesium alloy. The corrosion resistance was dramatically increased (86.9  kΩ·cm^2^) in comparison with the bare sample (0.2  kΩ·cm^2^). Mrad [28] compared the physicochemical and anti-corrosion properties of a pure PANI coating and a bi-layer fabrication consisting of an inner PANI layer and an external poly (γ-glycidoxypropyltrimethoxysilane (Poly (γ-GPTMS)) layer. The inner PANI layer was electrodeposited onto an AA2024-T3 substrate, whereas the external silane film was prepared using dip-coating technology. Results revealed that the synthesized PANI/poly (γ-GPTMS) coating achieved better adhesion, water repellency and corrosion resistance than the pure PANI. To sum up, multi-layer strategies are effective methods to overcome the deficiencies in corrosion resistance of pure PANI layers.

Although in our previous study [29] a well-established pure PANI coating was prepared by using CV technology in a benzoic acid electrolyte, the corrosion resistance still needs to be improved further. As far as we know, little research has been reported on the fabrication of a superhydrophobic PANI/silane composite coating using a specific silane formulation (TEOS/KH560/HDTMS), and the anti-corrosion performance of this hybrid coating still needs deeper investigation. In the present work, a superhydrophobic polyaniline/silane hybrid coating was obtained via a two-step electrodeposition method to protect mild steel against corrosion in a 3.5 wt.% NaCl solution. The morphology and chemical composition of the obtained PANI/silane hybrid were detected using FTIR, SEM and XPS. The superhydrophobic surface was verified using contact angle measurements. The anti-corrosion properties were evaluated using open circuit potential (E_ocp_), potentiodynamic polarization plots and electrochemical impedance spectroscopy (EIS). In addition, the corrosion protection mechanism of the synthesized composite coating was elucidated in detail.

## 2. Experimental

### 2.1. Materials

The low-carbon steel (Q235) used in this experiment was purchased from Shengtai New Materials Co., Ltd. (Dongguan, China) and was cut into specimens of 1 cm × 1 cm × 0.2 cm. Aniline, 3-glycidoxypropyltrimethoxysilane (KH560), tetraethyl orthosilicate (TEOS), hexatriemethoxysilane (HDTMS), and ethanol were bought from Shanghai Macklin Biochemical Technology Co., Ltd. (Shanghai, China) Lithium nitrate and benzoic acid (analytical-grade) were obtained from Sinopharm Chemical Reagent Co., Ltd. (Shanghai, China). The platinum mesh electrode and saturated calomel electrode (SCE) were bought from Shanghai Xianren Instrument Co., Ltd. (Shanghai, China). All reagents were used directly without further purification. The low-carbon steel specimens were encapsulated with an epoxy resin, exposing only a 1.0 cm^2^ working surface. Subsequently, they were sequentially ground with 120# to 1200# sandpapers, polished using 2.5 μm diamond abrasive particles, and finally ultrasonically cleaned in acetone and ethanol for 5 min each, followed by N_2_ blow-drying for subsequent use.

### 2.2. Fabrication of Superhydrophobic Pani/Silane Coating

**Figure 1:**
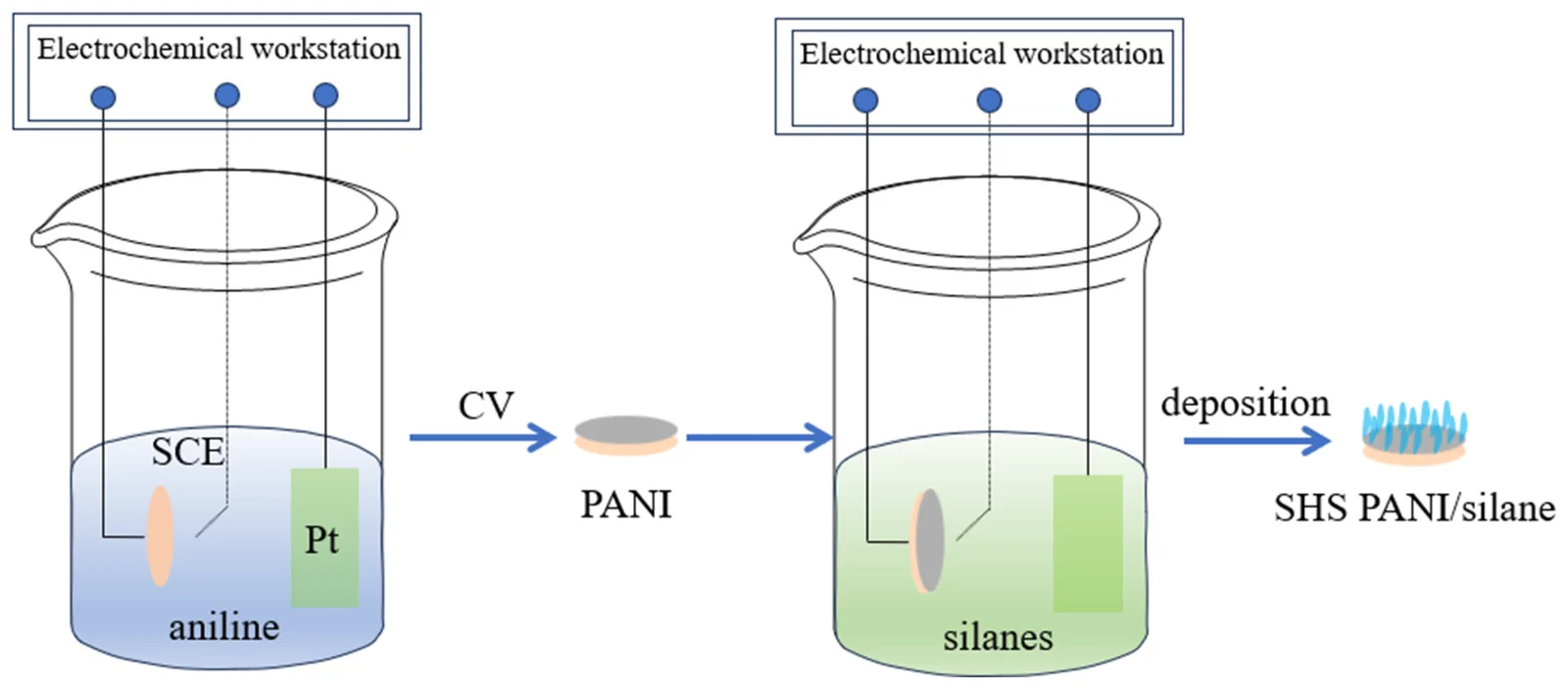
Scheme diagram for fabrication of superhydrophobic PANI/silane composite coating on Q235 mild steel substrate.

The superhydrophobic PANI/silane composite coating was fabricated on the Q235 substrate via a two-step electrochemical deposition method, and the preparation process is schematically depicted in Figure 1. Following typical procedure, 0.04 M benzoic acid, 0.15 M lithium nitrate, and 0.4 M aniline were dissolved into a mixed solvent that contained ethanol and de-ionized water in a volume ratio of 4:1, which was designated as Solution A. Then, a mixture of ethanol, de-ionized water, TEOS, KH560, and HDTMS with a volume ratio of 75:25:2:1:1 was prepared and the specific silane formulation was hydrolyzed under magnetic stirring at 40 °C for 30 h, designated as Solution B.

Step 1: The pretreated Q235 substrate, platinum mesh and a saturated calomel electrode (SCE) were utilized as the working electrode, counter electrode, and reference electrode, respectively. Electrochemical polymerization of a pure PANI coating on the Q235 mild steel surface was performed in Solution A via cyclic voltammetry technology [29]. The polymerization was conducted between −0.5 V and 1.6 V (vs. SCE) at a scan rate of 20 mV/s for 10 cycles.

Step 2: The pure-PANI-coating-covered Q235 substrate was immersed into the completely hydrolyzed Solution B, and deposition was carried out at a constant potential of −1.2 V (vs. SCE) for 15 min. After deposition, the specimens were rinsed with de-ionized water and dried in an oven at 120 °C for 2 h. A superhydrophobic film was constructed through the hydrolysis–condensation of a specific silane formulation (TEOS/KH560/HDTMS). The prepared superhydrophobic PANI/silane coating was labeled as SHS PANI/silane.

### 2.3. Structure Characterization

The thicknesses of the prepared pure PANI coating and SHS PANI/silane composite coating were measured using a Dektak 150 profilometer (Bruker, Karlsruhe, Germany) and were about 27.0 and 33.5 μm, respectively. The wettability of the designed coatings was examined using a JC2000D1 CA system. At least five positions on the pure PANI coating and SHS PANI/silane composite coating were tested and the corresponding standard deviations were 1.3° and 1.6°, respectively. The FTIR spectra were measured using a Nexus 670 infrared spectrometer (Thermo Nicolet Scientific, Madison, WI, USA) over the 4000–450 cm^−1^ wavenumber range. The surface morphology was observed using a Quanta FEG 450 scanning electron microscope (FEI Company, Hillsboro, OR, USA) at different magnifications. The chemical composition was characterized using an Escalab 250 Xi X-ray photoelectron spectroscopy (Thermo Nicolet Scientific). The adhesion of the fabricated coatings after 10 days of immersion in 3.5 wt.% NaCl solution was tested according to the ISO 2409 standard [30].

### 2.4. Electrochemical Experiments

Electrochemical experiments were carried out to evaluate the anti-corrosion ability of the designed pure PANI coating and SHS PANI/silane composite coating on a commercial electrochemical workstation (PARSTAT 4000A, AMETEK, Berwyn, IL, USA) in a 3.5 wt.% NaCl solution. The tests were conducted at a constant temperature of 25° using an aqueous thermostatic waterbath technique. A classic three-electrode electrochemical system, with platinum foil as the auxiliary electrode, an SCE as the reference electrode, and the coated and uncoated mild steel samples as the working electrodes, was applied as the working cell. EIS data were recorded in frequency ranges from 10^5^ to 10^−2^ Hz at the open circuit potential and a perturbation of 10 mV was applied. The potentiodynamic polarization plots were scanned from cathodic to anodic potentials at E_OCP_ ± 250 mV with a scanning rate of 0.5 mV/s. Each electrochemical measurement was conducted three times on independent specimens to ensure their reliability.

## 3. Results and Discussion

**Figure 2:**
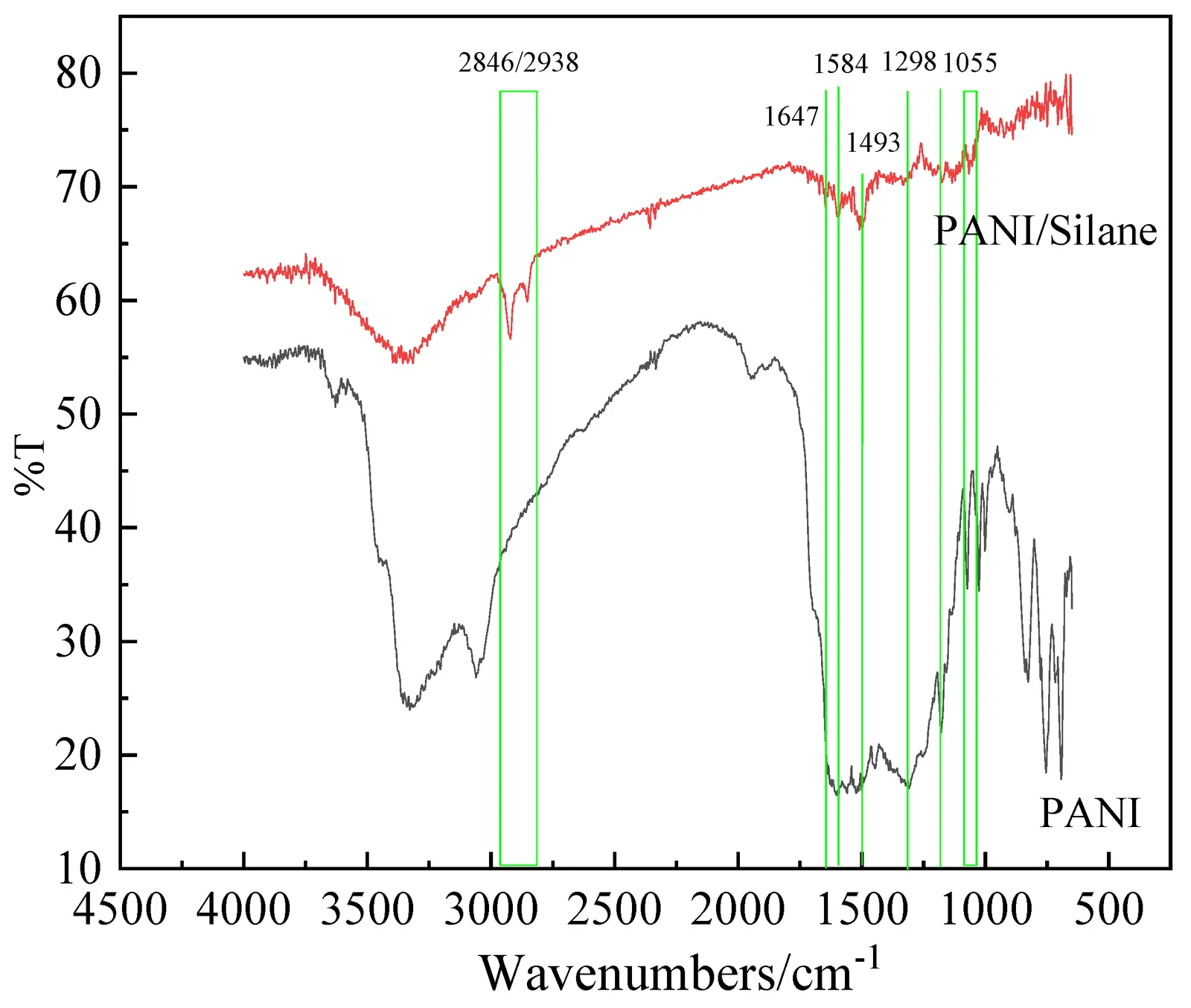
FTIR of prepared pure PANI and SHS PANI/silane coatings.

The FTIR spectra of the prepared pure PANI and SHS PANI/silane coatings are shown in Figure 2. For the electropolymerized pure PANI coating, the peaks ranging from 3050 cm^−1^ to 3350 cm^−1^ are ascribed to the symmetric and asymmetric stretching vibrations of NH and NH_2_ [12]. The characteristic peaks at 1584 cm^−1^ and 1493 cm^−1^ are assigned to the vibration of quinoid rings and benzenoid ring units, respectively, which suggest the emeraldine base form of the prepared pure PANI coating under this electropolymerization condition [29]. The broad peak at around 1298 cm^−1^ corresponds to the C–N stretching of a secondary aromatic amine, and another sharp peak at 1159 cm^−1^ is attributed to vibrations associated with the C–H of N=Q=N (Q, quinoid rings). These observations further show that the synthesized pure PANI coating was in an emeraldine state [31]. In the case of the SHS PANI/silane composite coating, the peaks at 2938 cm^−1^ and 2846 cm^−1^ are ascribed to the symmetric and asymmetric stretching vibrations of C–H bonds in the long-chain alkyl groups of HDTMS, indicating that HDTMS molecules were successfully grafted onto the prepared pure PANI coating surface. The strong peak at 1055 cm^−1^ is attributed to the vibration of Si–O–Si bonds, derived from the hydrolysis–condensation reactions of silanol groups [32]. The above results imply a successful modification of the underlying pure PANI layer with a silane film added on top.

**Figure 3:**
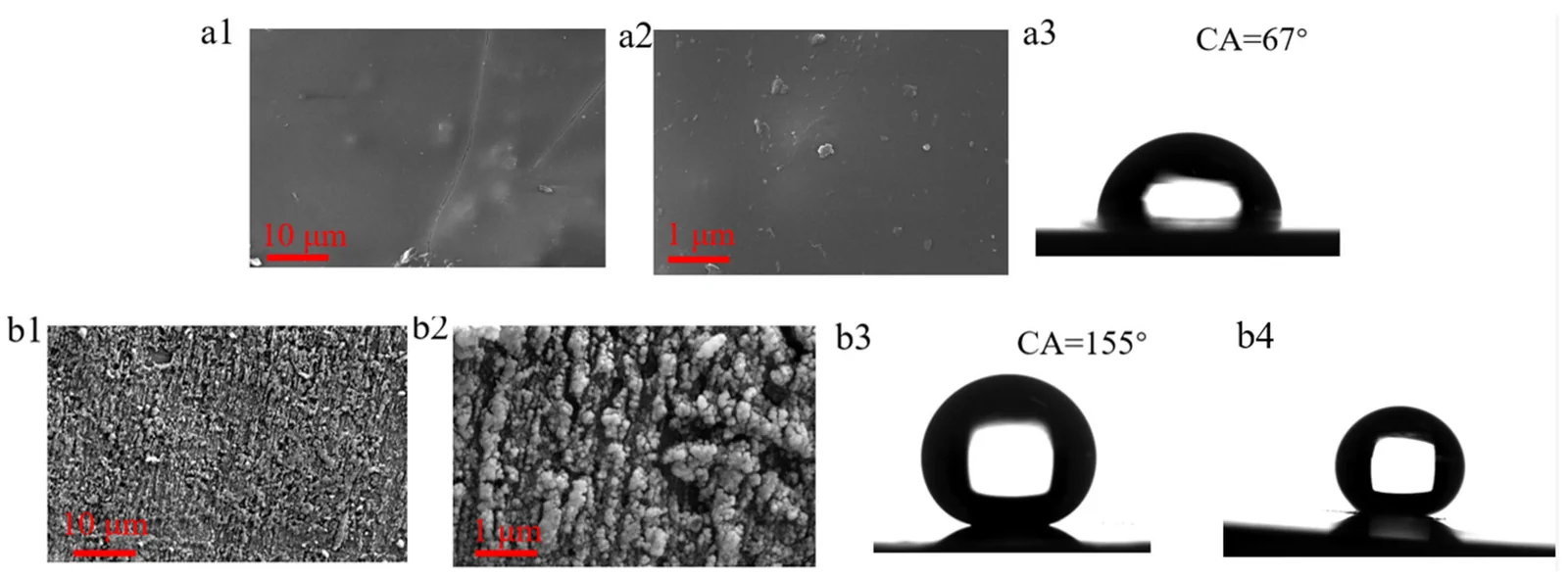
Surface morphology (**a1**), enlarged graph (**a2**) and CA (**a3**) of prepared pure PANI coating; surface morphology (**b1**), enlarged graph (**b2**), CA (**b3**) and sliding angle (**b4**) of prepared SHS PANI/silane coating.

Figure 3 presents the surface morphology and CA of the prepared pure PANI and SHS PANI/silane composite coatings. It is apparent that the surface of the prepared pure PANI coating exhibits a smooth and uniform structure (Figure 3(a1,a2)), and the CA was found to be about 67° (Figure 3(a3)), which suggests the hydrophilic nature of the synthesized emeraldine base PANI coating. In contrast, a hierarchical morphology of the SHS PANI/silane composite coating was observed with some nanoparticle accumulation and micron-scale protrusions (Figure 3(b1,b2)). These constituents are interconnected, forming a three-dimensional network-like porous structure. This morphology originates from the synergistic effect of the two-step deposition process: the underlying pure PANI layer provides good conductivity and cohesion, whereas the condensation of hydrolyzed silane monomers facilitates high surface roughness and low surface energy to produce a high CA value of 155° for the fabricated composite coating. According to the Cassie–Baxter model [33], the rough surface traps large amounts of air in the nano–microstructures and the water droplets on the surface can roll easily, leading to a small sliding angle of approximately 4.2°.

**Figure 4:**
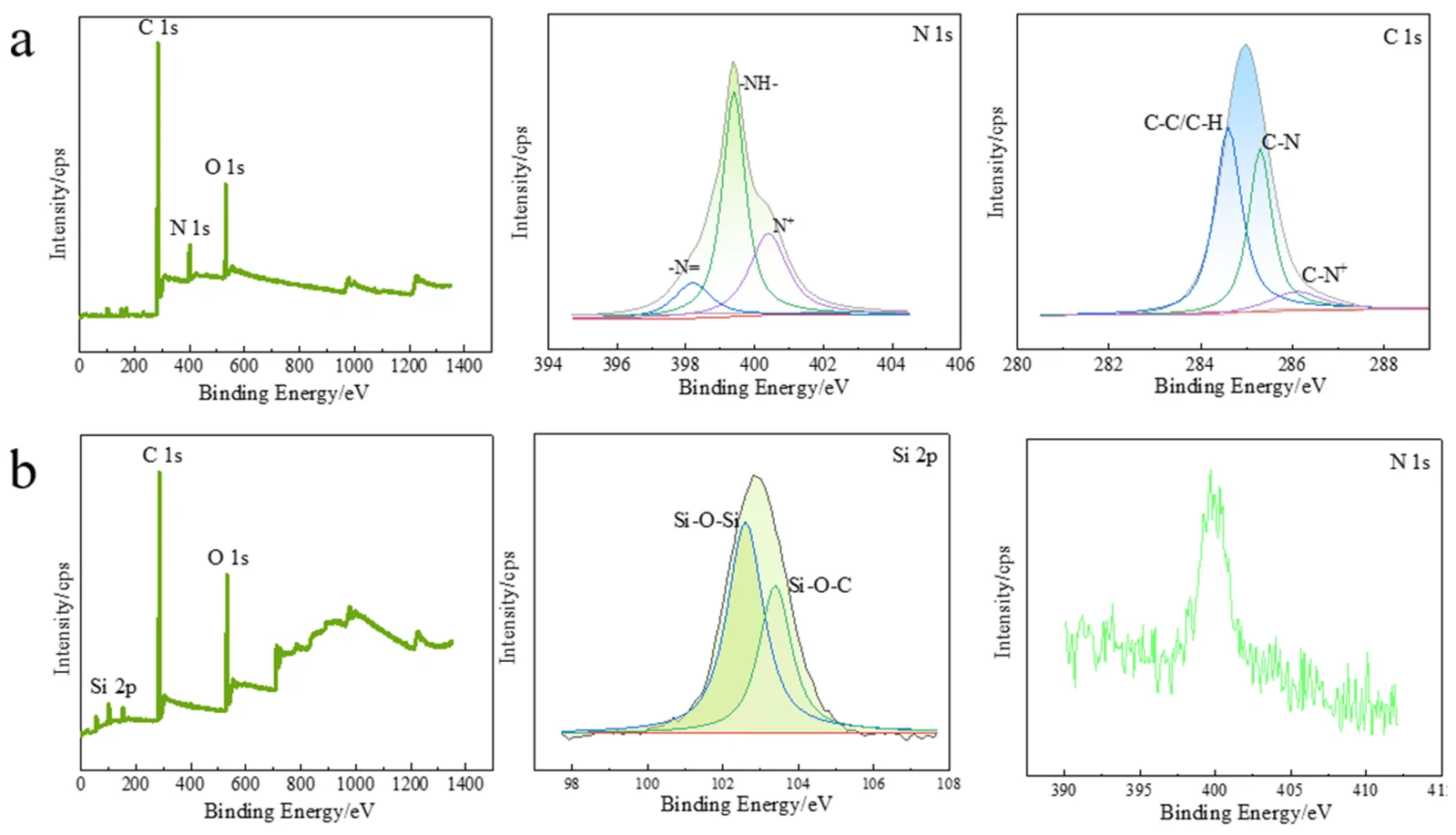
XPS spectra of prepared (**a**) pure PANI and (**b**) SHS PANI/silane coatings.

Figure 4 displays the XPS spectra of the prepared pure PANI and SHS PANI/silane composite coatings. Three elements, including C, N and O, can be detected for the prepared pure PANI coating. The N 1s spectrum contains three characteristic peaks [34]. The peaks at binding energies of 398.2 and 399.6 eV are ascribed to the neutral imine (–N=) and amine (–NH–) structures, respectively [31]. Another peak at a binding energy above 400 eV is related to the cation state of nitrogen (N^+^) [35]. The high-resolution C 1s spectrum shows three peaks at binding energies of 284.6 eV, 285.4 eV and 286.1 eV, which are assigned to C–C/C–H, C–N, and C–N^+^, respectively [36]. For the prepared SHS PANI/silane coating, due to the complete coverage by the top silane coating, the N element was hardly observed in the full spectrum. The Si 2p spectrum can be divided into two peaks at about 102.3 eV and 103.4 eV, which are attributed to the Si–O–Si and Si–O–C bonds, respectively [32]. The high-resolution N 1s spectrum suggests the presence of C–N bonds. The shift in the –NH– bond from 399.6 to 399.9 eV may result from the interaction between the underlying PANI layer and surface silane film. The above characterization results reveal that the surface silane film was successfully grafted onto the underlying PANI layer, and that its superhydrophobic nature and good barrier effect may promote enhanced corrosion protection by the prepared composite coating.

**Figure 5:**
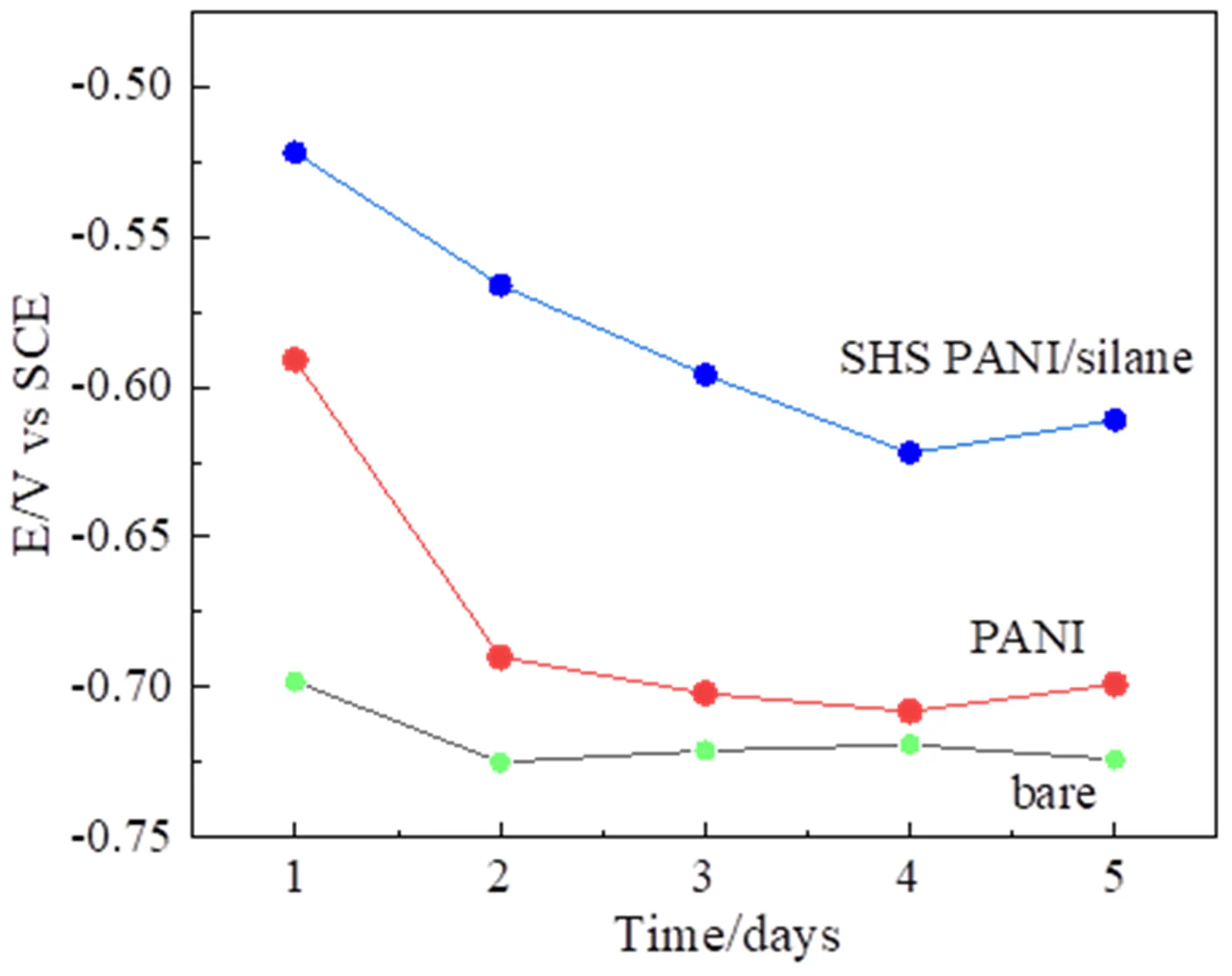
E_ocp_ of Q235 mild steel substrates, uncoated and with pure PANI and SHS PANI/silane coatings, in 3.5 wt.% NaCl solution over different durations.

E_ocp_ is an important criterion to evaluate corrosion protection ability. Figure 5 shows E_ocp_ values of Q235 mild steel substrates uncoated and with the prepared pure PANI and SHS PANI/silane coatings in a 3.5 wt.% NaCl solution at different durations. The value of E_ocp_ for the blank sample dropped to approximately −0.725 V (vs. SCE) after two days of immersion and remained relatively stable, indicating that the surface was rapidly activated and an electric double layer was formed. The initial potential of the prepared pure PANI coating was about −0.587 V, which showed a positive shift compared with the control. However, the potential decreased to a stable value close to −0.693 V within two days, suggesting that the electrolyte gradually permeated to the substrate interface through the pores and defects in the coating. In contrast, the initial potential of the fabricated SHS PANI/silane composite coating exhibited a significant positive shift, reaching −0.523 V, and the potential decay rate was much slower. The potential maintained a relatively high value after stabilization, which demonstrates that the top silane film effectively delayed electrolyte permeation.

**Figure 6:**
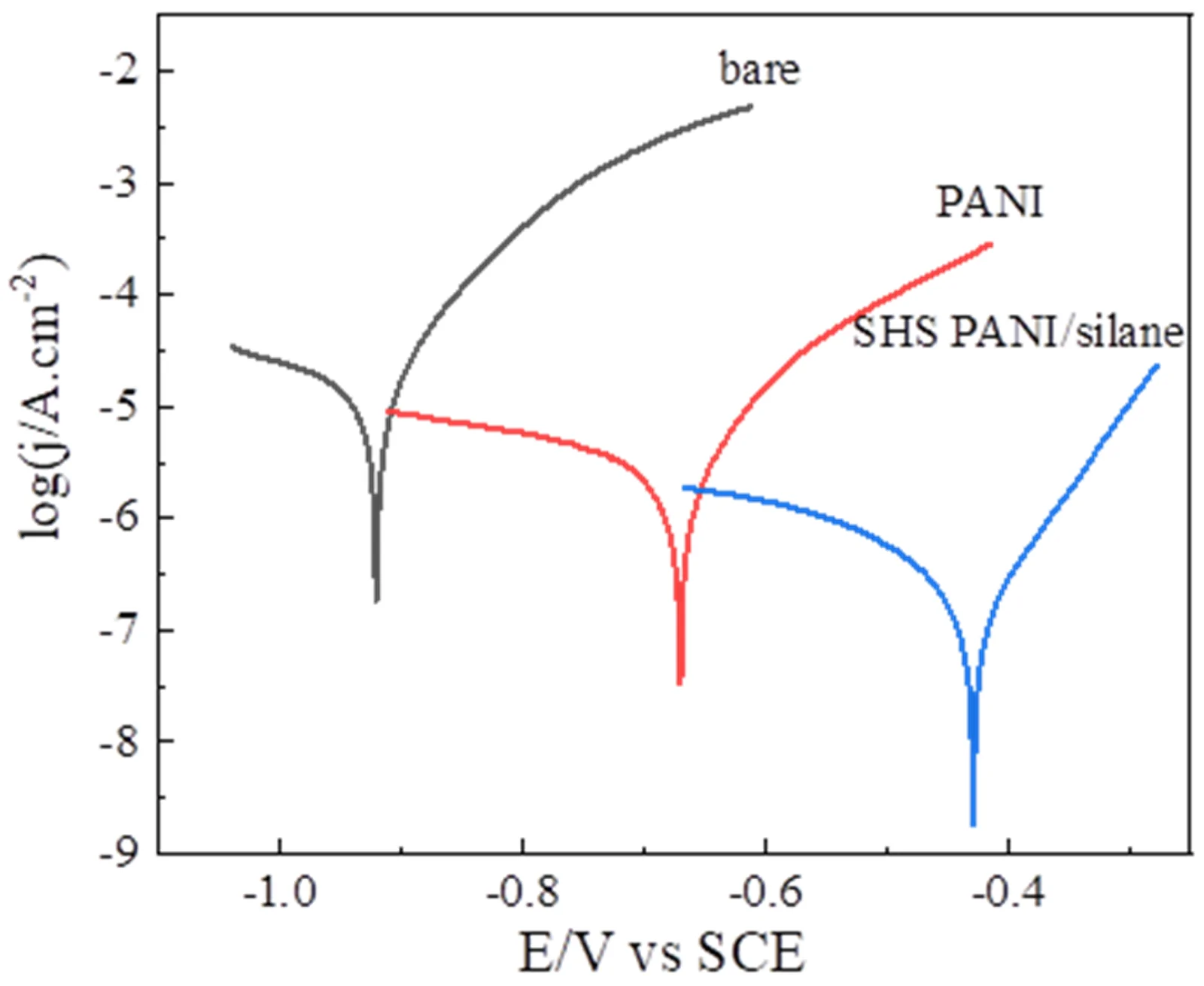
Potentiodynamic polarization curves of Q235 mild steel substrates, uncoated and with pure PANI and SHS PANI/silane coatings, in 3.5 wt.% NaCl solution.

Figure 6 presents the potentiodynamic polarization curves of Q235 mild steel substrates when left bare and coated with the pure PANI and SHS PANI/silane coatings in a 3.5 wt.% NaCl solution. The current densities of both the anodic and cathodic branches are considerably suppressed by the prepared pure PANI and SHS PANI/silane coatings, suggesting decreased corrosion rates of the anodic dissolution process ($Fe-2e^{-}\to {Fe}^{2+}$) and cathodic oxygen reduction reaction ($O_{2}+2H_{2}O\to {4OH}^{-}$). Meanwhile, the shape of the potentiodynamic polarization curve for the bare sample is almost the same as those of the samples covered by the pure PANI and SHS PANI/silane coatings, which means that the corrosion mechanism was not altered and the protection of the Q235 substrate in a 3.5 wt.% NaCl solution by the fabricated pure PANI and SHS PANI/silane composite coatings was achieved through blocking the active sites on the Q235 substrate surface. It is interesting that although the underlying PANI layer protects metal materials from corrosion through a passivation effect, no passivation domain can be observed in the potentiodynamic polarization curves. This result has also been reported by other researchers [12,37], and may be ascribed to the limited potential window. Oppositely, Wang [38] suggested an obvious passive effect when enlarging the positive polarization potential to 0 V vs. SCE for pure PANI and PVP-PANI coatings. The electrochemical parameters, including corrosion potential (*E_corr_*), corrosion current density (*j_corr_*), cathodic slope (*β_c_*) and anodic Tafel slope (*β_a_*), were obtained from an extrapolation of Tafel lines and the results are summarized in Table 1. The corrosion protection efficiency (η%) was calculated as follows [39], and the values of η% are also listed in Table 1:

(1) η=1−jcorrcjcorrbare

where $j_{corr}^{c}$ and $j_{corr}^{bare}$ represent the corrosion current densities for the coated and uncoated samples, respectively.

As shown in Table 1, the blank sample exhibited the highest corrosion current density of 34.62 μA/cm^2^, whereas the corrosion current densities of the samples with the prepared pure PANI coating and SHS PANI/silane composite coating were 5.694 μA/cm^2^ and 0.142 μA/cm^2^, respectively. Compared with the blank carbon steel substrate, the sample with an electropolymerized pure PANI coating showed a significant positive shift in E_corr_. Similarly, the sample with the fabricated SHS PANI/silane composite coating showed a notable positive movement in E_corr_ relative to the pure PANI coating. This observation reveals that the SHS PANI/silane-coated sample possessed the best thermodynamic stability. Furthermore, the SHS PANI/silane hybrid exhibited a high corrosion protection efficiency of 99.6%.

**Figure 7:**
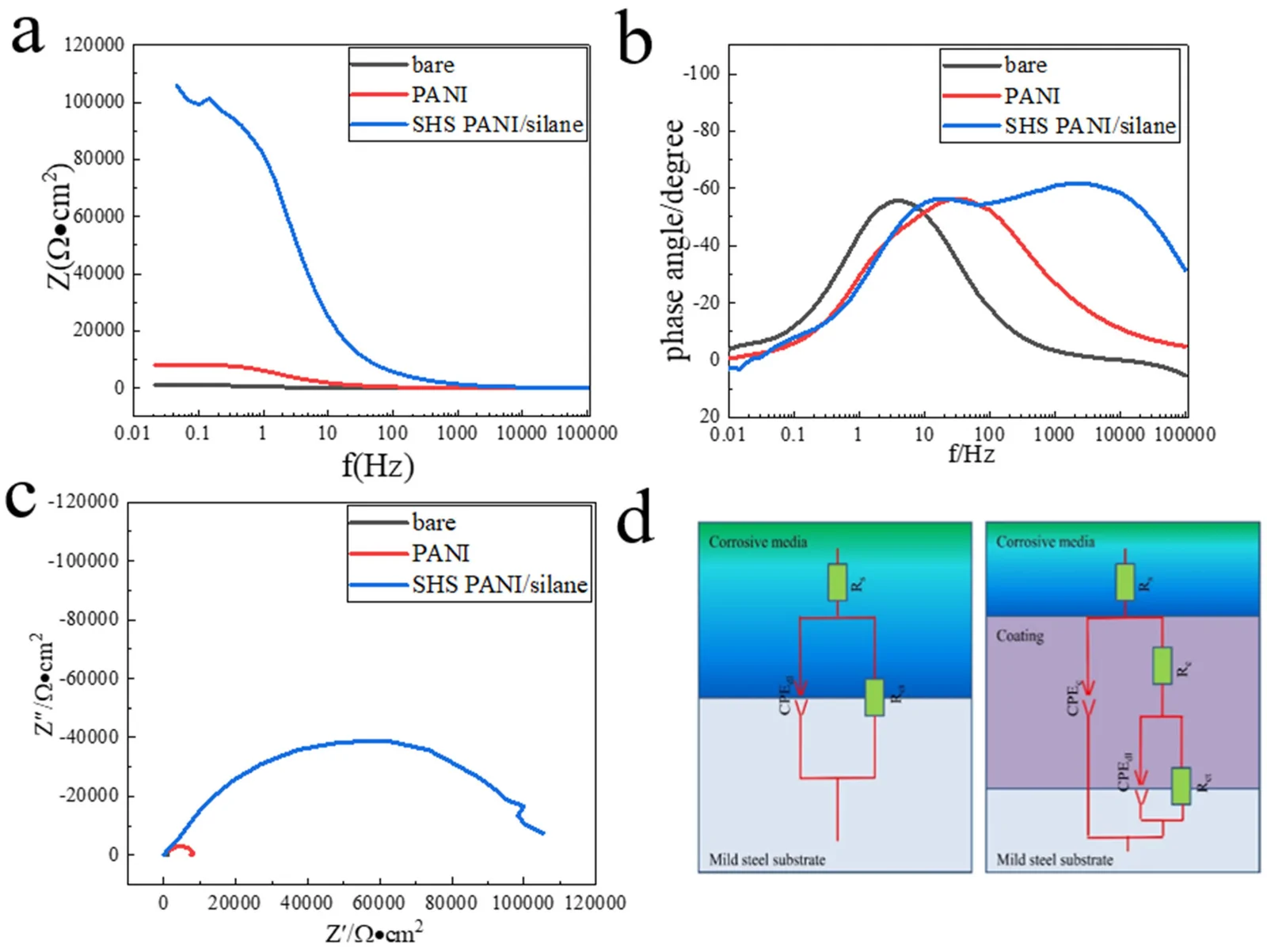
(**a**,**b**) Bode plots; (**c**) Nyquist plots of Q235 mild steel substrates, uncoated and with pure PANI and SHS PANI/silane coatings, in 3.5 wt.% NaCl solution; (**d**) equivalent electric circuits.

Figure 7a–c show the Bode and Nyquist plots of a bare Q235 mild steel substrate and two covered with pure PANI and SHS PANI/silane coatings in a 3.5 wt.% NaCl solution. The pure PANI- and SHS PANI/silane-coated samples exhibited larger impedance moduli than the control, revealing lower corrosion rates due to the protective layers. Meanwhile, the diameter of the prepared SHS PANI/silane-coated sample was larger than that of the pure PANI-coated one, indicating that the SHS PANI/silane coating possessed better anti-corrosion properties. This phenomenon suggests that the corrosion protection ability of the pure PANI coating was significantly enhanced by the grafted top silane coating. In the Bode plots, the bare Q235 specimen shows one phase peak, whereas the pure PANI- and SHS PANI/silane-coated samples consist of one main peak in the high-frequency domain and another shoulder peak in the middle frequency range, which correlate with established knowledge on surface coating and substrate/electrolyte interface reactions, respectively [40]. Xu [41] also reported a two-phase-angle-peak feature in phase angle–frequency plots when investigating the anti-corrosion performance of polyaniline–silica coating and suggested that these two peaks were correlated with the external layer capacitor and interface constant-phase capacitance, respectively. Therefore, two kinds of equivalent electric circuits (EECs), as shown in Figure 7d, were utilized to fit the experimental EIS data using Z-view 3.3 software. In the equivalent electric circuits, R_s_, R_ct_ and R_c_ represent the solution resistance, charge transfer resistance and coating resistance, respectively. CPE_dl_ and CPE_c_ are the double electric layer capacitance and coating capacitance, respectively. The obtained values of EIS parameters are shown in Table 2. The polarization resistance (R_p_) and coating protection efficiency (PE%) were calculated by the following equations [42,43]:

(2) $$R_{P}=R_{c}+R_{ct}$$

(3) $$PE\%=\frac{R_{P}^{c}-R_{p}^{bare}}{R_{P}^{c}}$$

where $R_{P}^{c}$ and $R_{p}^{bare}$ represent the polarization resistances for the coated and uncoated samples, respectively. The values of R_p_ and PE% are also summarized in Table 2. The value of R_ct_ increases by 87 and 9 times in comparison with the bare sample for the samples coated with the fabricated SHS PANI/silane and pure PANI coatings, respectively. The value of R_c_ for the SHS PANI/silane-coated sample was 14.7 kΩ·cm^2^, which increased by over one order of magnitude compared to the pure PANI-coated sample, revealing a remarkably improved corrosion protection ability of the PANI layer with the addition of the modified top silane coating. It is worth noting that the values of CPE_dl_ present the following trend—bare > pure PANI > SHS PANI/silane—which implies that the prepared SHS PANI/silane composite coating resulted in the weakest corrosion reaction at the interface. The PE% of the SHS PANI/silane-coated sample was 99.0%, confirming an excellent anti-corrosion performance.

**Figure 8:**
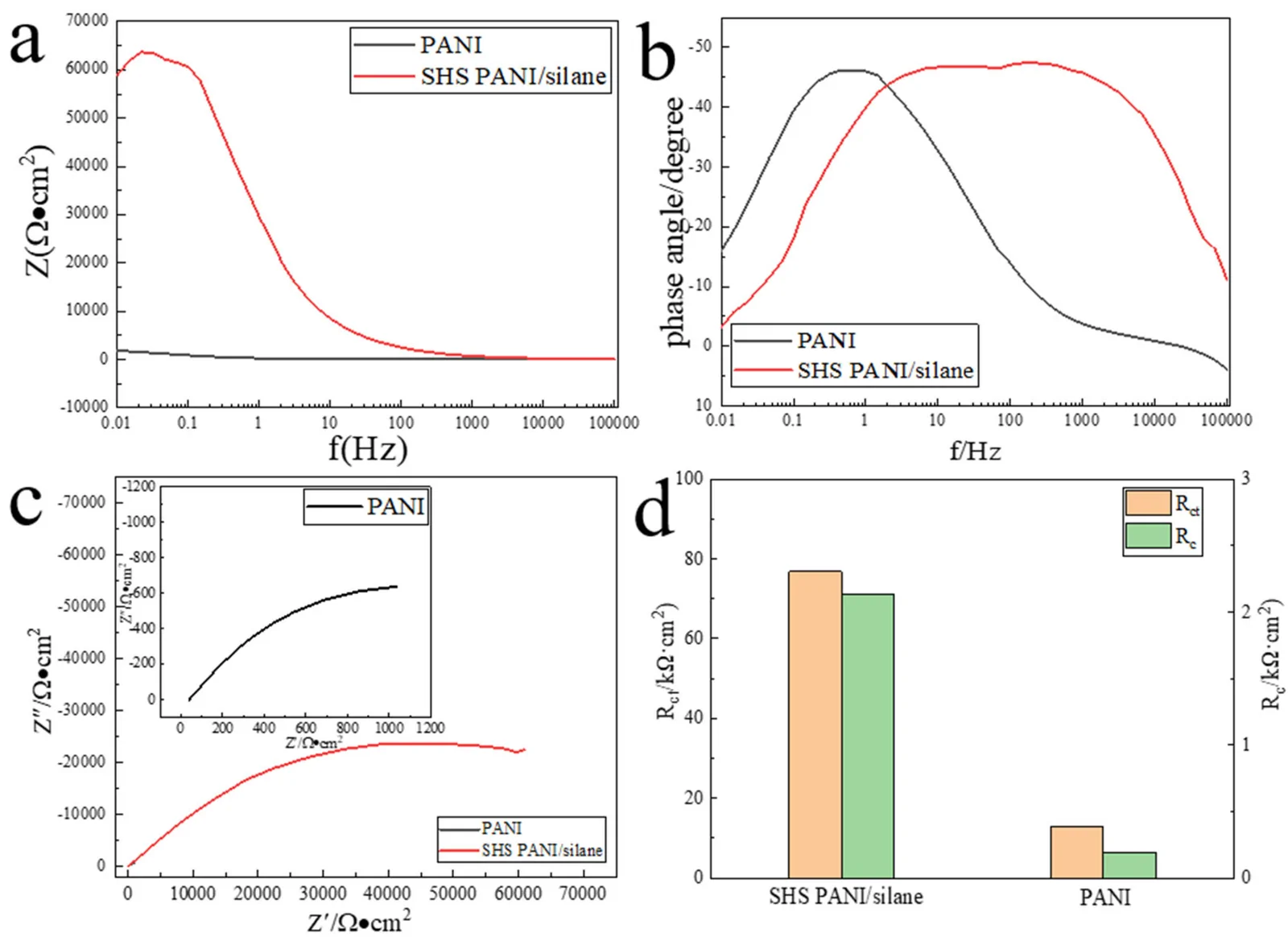
(**a**,**b**) Bode plots; (**c**) Nyquist plots and (**d**) fitted values of R_ct_ and R_c_ of prepared (**a**) pure PANI and (**b**) SHS PANI/silane coatings after 10 days of immersion.

Figure 8 presents the EIS diagrams and corresponding fitted parameters of pure PANI- and SHS PANI/silane-coated samples after 10 days of immersion in a 3.5 wt.% NaCl solution. It is clear that the impedance modulus decreased for both pure PANI- and SHS PANI/silane-coated samples after 10 days of exposure, which suggests that the coatings’ anti-corrosion ability was weakened. The EIS parameters were obtained from fitting the EIS experimental data according to the EEC in Figure 7d, and the values were plotted as shown in Figure 8d. The values of R_ct_ decreased from 93.6 kΩ·cm^2^ and 9.74 kΩ·cm^2^ to 76.8 kΩ·cm^2^ and 2.13 kΩ·cm^2^, whereas the values of R_c_ decreased from 14.7 kΩ·cm^2^ and 0.81 kΩ·cm^2^ to 12.9 kΩ·cm^2^ and 0.19 kΩ·cm^2^ for the samples with SHS PANI/silane and pure PANI coatings, respectively. This finding demonstrates that the fabricated SHS PANI/silane composite coating caused smaller reductions in R_ct_ and R_c_ and presented a much better chemical stability compared to the pure PANI coating.

**Figure 9:**
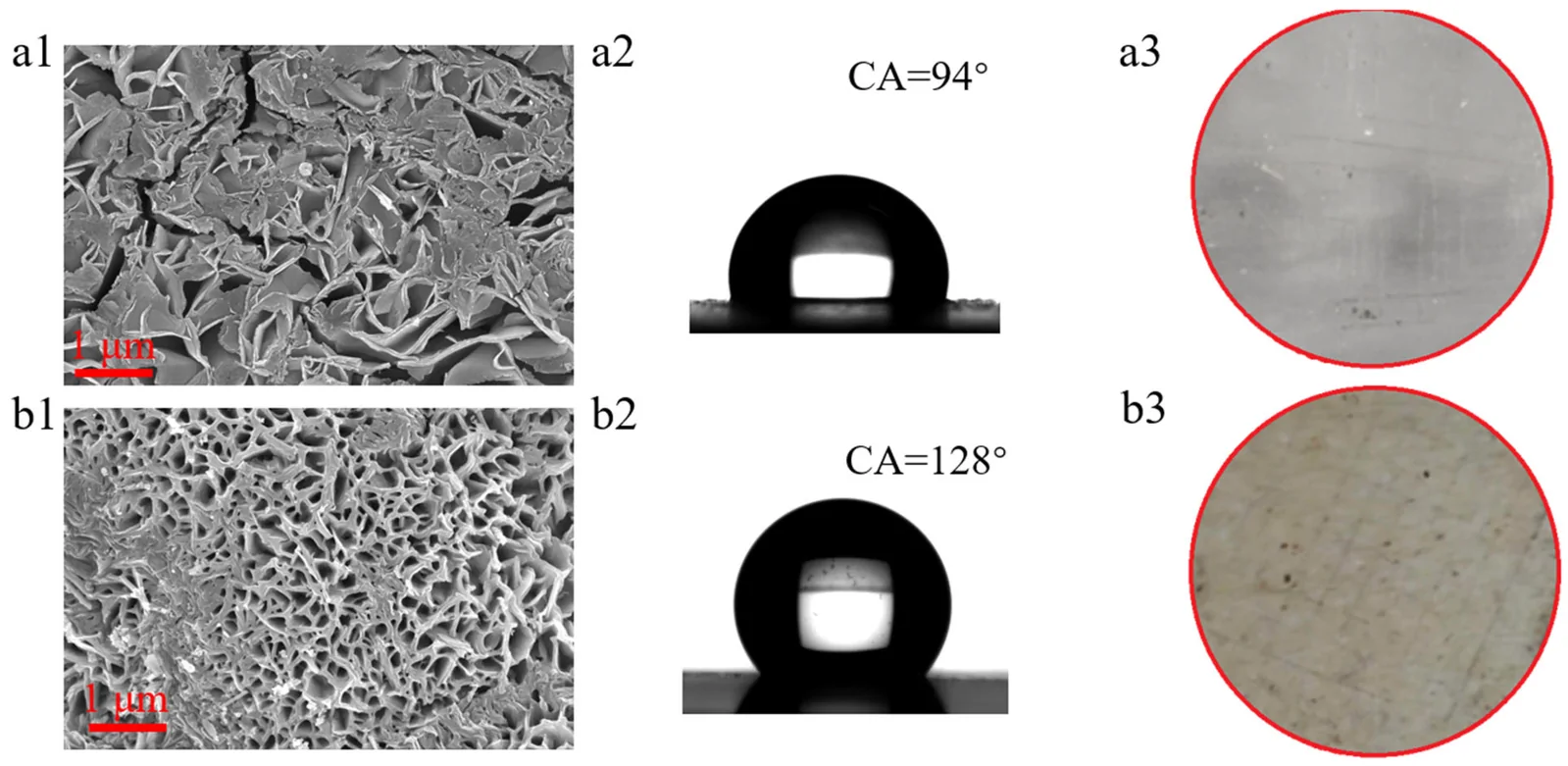
Surface morphology (**a1**), CA (**a2**) and adhesion (**a3**) of pure PANI coating after 10 days of immersion; surface morphology (**b1**),CA (**b2**) and adhesion (**b3**) of SHS PANI/silane coating after 10 days of immersion.

The surface morphology, CA and adhesion of the pure PANI and SHS PANI/silane coatings after 10 days of immersion are shown in Figure 9. The initial smooth and uniform structure of the pure PANI coating (Figure 3(a1)) was dramatically destroyed and many defects and holes can be observed across the whole surface, which produced a rougher structure and caused a slightly higher CA. The defects in the pure PANI coating considerably promote the penetration of aggressive ions to the substrate/coating interface and then lead to significant reductions in R_c_ and R_ct._ However, in the case of the SHS PANI/silane coating, the hierarchical micro–nanostructure was less damaged. The CA decreased from 155° to 128° due to a weak degradation of the top silane film. In fact, the superhydrophobic surface can be destroyed by long-term exposure to corrosive environments, which has also been reported by other researchers [44,45]. For example, Abbasi [46] observed a sharp decrease in contact angle from 149° to 79° after immersing a superhydrophobic silane coating for 3 days in a 3.5 wt.% NaCl solution. In the adhesion test results, the pure PANI coating showed almost no peeling after the adhesion test, with only scratches and indentations remaining, indicating that the adhesion strength of the prepared pure PANI coating is close to ISO adhesion grade 0. Meanwhile, for the SHS PANI/silane composite coating, only a minimal amount of peeling occurred after tape adhesion and tearing, suggesting that this hybrid coating also possesses good adhesion, reaching an ISO adhesion grade of level 1.

The excellent anti-corrosion properties of the fabricated SHS PANI/silane composite coating lies in the following aspects. Firstly, the superhydrophobic nature of the surface silane film facilitates the repelling of aggressive species from the composite coating, which results in a remarkable delay of the corrosion process [47]. This result is in significant accordance with other reports [48,49]. For example, Zhou [50] suggested that their prepared superhydrophobic Ni/Mn-TiO_2_ composite coating on carbon steel resulted in a much higher corrosion resistance than that of untreated carbon steel. Secondly, both the surface SHS silane film and underlying PANI layer act as effective physical barriers, which inhibit the penetration and diffusion of corrosive media toward the mild steel substrate interface. It is known that the PANI layer can supply anodic protection to iron-based metal materials due to its higher reduction/oxidation potential than that of iron, which consequently maintains the passive state of the metal substrate [51]. The reaction that occurs at the interface between the PANI layer and mild steel substrate is as follows:

(4) $$Fe+\frac{n}{m}{PANI}^{m+}+{yH}_{2}O\to {Fe(OH)}_{y}^{\left(n-y\right)+}+\frac{n}{m}{PANI}^{0}+{yH}^{+}$$

(5) $${\frac{m}{4}O}_{2}+{\frac{m}{2}H}_{2}O+{PANI}^{0}\to {PANI}^{m+}+{mOH}^{-}$$

Reaction 4 displays the electrochemical process taking place at the mild steel/PANI layer interface, causing the oxidation of the substrate metal to form a dense oxide film on the mild steel substrate surface. Reaction 5 describes the oxidation of a reduced PANI layer to regenerate the oxidized PANI at the coating–electrolyte interface. As a result, the PANI under-layer served as a redox mediator between the mild steel substrate and corrosive electrolyte and maintained the mild steel substrate in a passive state, which further restrained the corrosion process [52].

Comparing our results to previous studies, the SHS PANI/silane composite coating prepared through a facile two-step electrodeposition strategy in the present work exhibited excellent corrosion protection properties (Table 3) and can be applied as a potential candidate for protective coatings in industrial applications.

## 4. Conclusions

In this work, a superhydrophobic polyaniline (PANI)/silane composite coating was prepared on a mild steel substrate using a two-step electrodeposition strategy. The first step consists of creating an adherent pure PANI coating using a cyclic voltammetry method and the second step consists of fabricating a superhydrophobic surface using electrochemical-assisted silanization. The prepared PANI/silane composite coating showed a high contact angle of 155° and low sliding angle of 4.2°. Electrochemical measurements demonstrated that the corrosion current density of PANI/silane-coated mild steel decreased by approximately three orders of magnitude compared to a blank sample, and the corrosion protection efficiency was 99.6%. Moreover, the prepared PANI/silane hybrid coating exhibited good chemical stability and strong adhesion after 10 days of corrosion in 3.5 wt.% NaCl solution, which was due to the synergistic effect between the underlying PANI layer and top silane film. The impressive corrosion resistance and adhesion of the fabricated PANI/silane composite coating mean that this work presents a controllable and large-scale preparation strategy for anti-corrosion application.

## Figures and Tables

**Table 1:** Potentiodynamic polarization parameters for Q235 mild steel substrates, uncoated and pure PANI and SHS PANI/silane coatings, in 3.5 wt.% NaCl solution.

Condition	E_corr._ (V)	j_corr._ (μA/cm^2^)	−*β_c_* (mV/dec)	*β_a_* (mV/dec)	ƞ%
bare	−0.912 ± 0.024	34.62 ± 0.95	268 ± 8.62	131 ± 4.25	-
PANI	−0.676 ± 0.016	5.694 ± 0.163	282 ± 7.86	144 ± 4.78	83.6
SHS PANI/silane	−0.433 ± 0.015	0.142 ± 0.015	237 ± 8.13	115 ± 3.49	99.6

**Table 2:** EIS parameters of Q235 substrates, uncoated and with PANI and SHS PANI/silane coatings, in 3.5 wt.% NaCl solution.

Condition	R_ct_ kΩ·cm^2^	CPE_dl_ Ω^−1^·cm^−2^·s^n^ × 10^−5^	n_dl_	R_c_ kΩ·cm^2^	CPE_c_ Ω^−1^·cm^−2^·s^n^ × 10^−5^	n_c_	R_p_ kΩ·cm^2^	*χ* ^2^	PE%
bare	1.07 ± 0.03	3.03 ± 0.06	0.82 ± 0.02	-	-	-	1.07	0.019	-
PANI	9.74 ± 0.26	1.26 ± 0.04	0.87 ± 0.03	0.81 ± 0.02	1.02 ± 0.03	0.73 ± 0.02	10.55	0.012	89.9%
SHS PANI/silane	93.6 ± 3.15	0.11 ± 0.003	0.89 ± 0.03	14.7 ± 0.46	0.43 ± 0.01	0.75 ± 0.02	108.3	0.016	99.0%

**Table 3:** Comparison of anti-corrosion performance of some conducting-polymer/silane composite coatings.

Sample	Substrate	Initial IE%	Long-Term IE%	Ref.
polyaniline/(TEOS/KH560/HDTMS)	Q235 mild steel	99.6	98.8 (10 days)	this work
polyaniline/(TEOS/VTES)/Ni	AZ91	99.7	98.3 (7 days)	[27]
polyaniline//poly(γ-GPTMS)	AA2024-T3	99.3	97.9 (7 days)	[28]
polyaniline/KH560	AZ31	99.6	-	[53]

## Data Availability

Data will be made available on request. The data presented in this study are available on request from the corresponding author.
